# Besides Pathology: Long Non-Coding RNA in Cell and Tissue Homeostasis

**DOI:** 10.3390/ncrna4010003

**Published:** 2018-01-30

**Authors:** Amanda Salviano-Silva, Sara Cristina Lobo-Alves, Rodrigo Coutinho de Almeida, Danielle Malheiros, Maria Luiza Petzl-Erler

**Affiliations:** Laboratory of Human Molecular Genetics, Department of Genetics, Universidade Federal do Paraná, Curitiba 81531-980, Caixa Postal 19071, Brazil; amandasalviano92@gmail.com (A.S.-S.); saralobo5@yahoo.com.br (S.C.L.-A.); rodrigocout@gmail.com (R.C.d.A.); mfdani@yahoo.com.br (D.M.)

**Keywords:** long non-coding RNA, homeostasis, physiological regulatory mechanisms, gene expression, gene regulation, transcriptome

## Abstract

A significant proportion of mammalian genomes corresponds to genes that transcribe long non-coding RNAs (lncRNAs). Throughout the last decade, the number of studies concerning the roles played by lncRNAs in different biological processes has increased considerably. This intense interest in lncRNAs has produced a major shift in our understanding of gene and genome regulation and structure. It became apparent that lncRNAs regulate gene expression through several mechanisms. These RNAs function as transcriptional or post-transcriptional regulators through binding to histone-modifying complexes, to DNA, to transcription factors and other DNA binding proteins, to RNA polymerase II, to mRNA, or through the modulation of microRNA or enzyme function. Often, the lncRNA transcription itself rather than the lncRNA product appears to be regulatory. In this review, we highlight studies identifying lncRNAs in the homeostasis of various cell and tissue types or demonstrating their effects in the expression of protein-coding or other non-coding RNA genes.

## 1. Introduction

Until a few years ago, non-coding RNAs (ncRNA) were an unexplored component of the transcriptome considered as transcriptional noise. The only well-characterized ncRNAs were the ribosomal RNAs (rRNA), transfer RNAs (tRNA), and small RNAs involved in the processing of primary transcripts, with the messenger RNAs (mRNA) being considered as the most numerous and diversified class of RNAs. However, technological advances, including large-scale sequencing and bioinformatics tools, revealed that ncRNAs are a major part of the total set of transcripts of the human genome [1,2]. These findings aroused interest in studying ncRNAs and, among them, long non-coding RNAs (lncRNAs).

**Figure 1:**
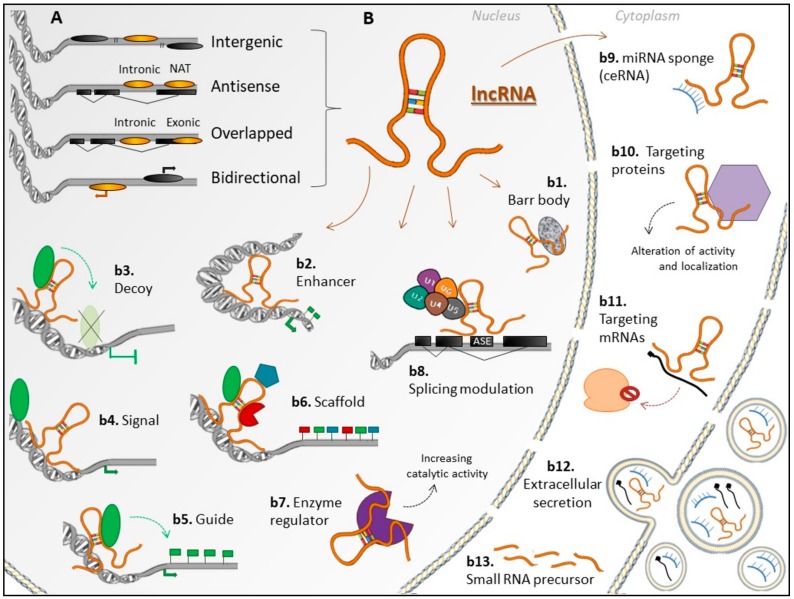
Genomic location relative to protein-coding genes, and regulatory mechanisms of long non-coding RNAs (lncRNAs) in the nucleus, cytoplasm, and extracellular compartments. (**A**) Nomenclature of lncRNA genes (gold ellipses), according to their genomic location relative to the nearest coding gene (black ellipses) and/or to exons of coding genes (black rectangles). (**B**) lncRNAs regulatory mechanisms: (b1) lncRNA *Xist*, as a component of Barr body in females; (b2) acting as enhancers, inducing transcription in *cis* or in *trans*; (b3) a decoy to regulatory proteins, such as transcription factors and chromatin modifiers, blocking their binding to DNA; (b4) as molecular signals, activating or silencing gene expression through signaling to regulatory pathways; (b5) Guiding proteins (in general, chromatin modifiers) to specific target sites; (b6) as scaffolds, binding different proteins and forming ribonucleoprotein (RNP) complexes, which also affect gene expression; (b7) interacting with enzymes, such as kinases, regulating/enhancing their catalytic activity and altering their signalization; (b8) modulating alternative splicing of primary transcripts; (b9) as competing endogenous RNA (ceRNA), serving as a sponge for microRNAs (miRNAs), blocking their effect; (b10) targeting proteins, forming molecular complexes which can block or induce functional effects, or even alter their location in the cell; (b11) targeting messenger RNAs (mRNAs), inhibiting their translation in ribosomes. In addition, lncRNAs can be (b12) transferred to other cells by extracellular vesicles (EVs), where they can produce effects; (b13) precursors of miRNAs and other regulatory small RNA. An lncRNA can act by multiple regulatory mechanisms, in both the nucleus and/or in the cytoplasm. The b12 itself is not exactly a regulatory feature, however, the release of these functional lncRNAs through EVs is a way of regulating genes, RNAs, or proteins in other tissues. ASE—alternatively spliced exon.

Long non-coding RNAs are defined as a heterogeneous class of ncRNAs longer than 200 nucleotides [1]. These transcripts are located predominantly in the nucleus and secondarily in the cytoplasm, being mostly polyadenylated and transcribed by RNA polymerase II. Long non-coding RNA genes may present tissue- and cell-specific expression and differ from mRNA genes by having longer and fewer exons [3]. According to the genomic position relative to the nearest coding gene, lncRNA genes can be categorized as intergenic (lincRNAs), bidirectional (divergent), sense or overlapped (intronic or exonic), and antisense (intronic or NAT, natural antisense transcript) [1] (Figure 1A). According to regulatory mechanisms on the expression of target genes or on other molecules, lncRNAs can act as signals (e.g., *Xist*), decoys (e.g., *Gas5, MALAT1*), scaffolds (e.g., *NEAT1*), guides (e.g., *HOTAIR*, *linc-p21*) [4,5], competing endogenous RNAs (ceRNAs) (e.g., *linc-MD1*) [6], and others. Moreover, their action as regulators of enzyme function was recently described [7,8]. Long non-coding RNAs can interact with enzymes and modulate their activities, as exemplified by *NBR2* that directly interacts with AMPK and promotes its kinase activity under energy stress [7] (Figure 1B).

Many studies have demonstrated the involvement of lncRNAs in processes of prime relevance for homeostasis [5]. One of the first lncRNAs studied, even before the recognition of this class of transcripts, was the *X inactivation-specific transcript* (*Xist*), located in the X inactivation center region and involved with X chromosome inactivation in mammalian females [9,10]. *Xist* expression is inhibited in the active X chromosome by another lncRNA, antisense to *Xist* promoter, called *Tsix* [11]. Overall, lncRNAs are known to be involved in gene expression regulation—at the transcriptional and post-transcriptional levels, by epigenetic or other mechanisms, such as interfering with the recruitment of RNA polymerase II or inducing chromatin remodeling. Furthermore, they participate in genomic imprinting; in nuclear and cytoplasmic trafficking; in protein localization and activity; and in interaction with miRNAs, among other processes (reviewed in [12]). In addition, they can be further processed to small ncRNAs [13] or even encode functional micropeptides [14,15]. However, little is known about how these transcripts control gene expression.

Long non-coding RNAs are strictly regulated [16,17] and participate in or are products of many biological processes [18,19]. Mutations in the primary sequence of lncRNAs, as well as aberrant variations of their expression, have been associated with several disorders, pointing to their potential as disease biomarkers [20]. Therefore, lncRNAs have been largely studied in different tissues’ homeostasis and pathology to understand their physiological effects and the consequences of their deregulation in complex diseases.

We performed an extensive search of the literature for articles presenting data about lncRNAs involved in the homeostasis of different tissues and cell types. Some of the lncRNA play fundamental roles in various tissues, while others present a tissue-specific expression pattern. We present the information by cell or tissue type throughout this review.

## 2. Long non-coding RNAs: Expression Patterns in Tissues or Cell Types

Long non-coding RNAs are strictly regulated and many present cell-specific expression, substantiating their crucial role in physiological mechanisms [1,3,21]. In the following, we summarized what is currently known about lncRNA expression among cell development and differentiation, and in specific pathways (more details in Table S1).

### 2.1. Hematopoietic Cells

Ontogenesis of blood cells from hematopoietic stem cells (HSCs) occurs throughout the whole individual’s life and is highly controlled by transcription factors and non-coding RNA. Circulating blood, where most of these cells are found, is easy to acquire and to work with, being routinely used in molecular studies. Yet, some authors analyzed bone marrow and thymus to understand early stages of hematopoiesis and the development of the different cell lineages.

The lincRNA *H19* (also known as *imprinted maternally expressed transcript*) is one of the most abundant and conserved mammalian noncoding transcripts, and a key regulator of differentiation of various cell and tissue types, as will also be seen in other sections of this review. The *H19* lincRNA is a transcript of the *H19/IGF2* genomic imprinted cluster. While *H19* is transcribed from the maternally-inherited locus, the mRNA for IGF2 (insulin-like growth factor II) is transcribed from the paternally-inherited locus. During murine hematopoiesis, the growth-restricting lincRNA *H19* was downregulated in HSCs before their proliferation and upregulated in long-term HSCs. *H19* is localized downstream of *Igf2* in the *H19/Igf2* locus. Both genes are co-expressed and have an antagonic effect on cell proliferation during hematopoiesis [22]. *H19* also inhibits HSC activation and proliferation, serving as a precursor of miR-675, a miRNA that targets the insulin-like growth factor 1 receptor (*Igf1r*) in placenta [23]. In another study, among the 323 novel lncRNAs identified in murine HSC RNA sequencing, 159 were HSC-enriched or likely HSC-specific (lncHSCs), in comparison with the transcriptome of differentiated B cells and granulocytes. Among these 159 lncRNAs, *lncHSC-1* was identified as involved in myeloid differentiation, and *lncHSC-2* as involved in HSC self-renewal and T cell differentiation. In addition, *lncHSC-2* is enriched with target sites for important hematopoietic-specific transcription factors, especially E2A [24].

**Figure 2:**
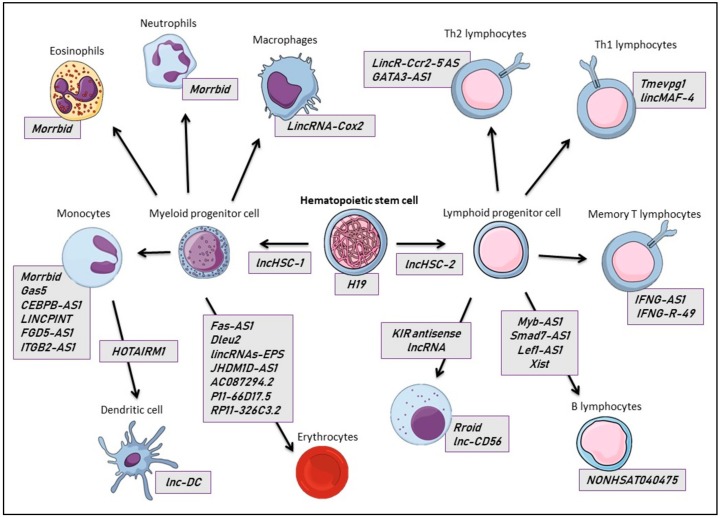
Long non-coding RNAs described in the physiology of mature and progenitor hematopoietic cells, derived from myeloid (**left**) and lymphoid (**right**) differentiation from a hematopoietic stem cell (HSC), in which the lncRNA *H19* plays a central role. In the grey rectangles are listed the lncRNAs specifically or differentially expressed in each cell type (rectangles at the side of cells), or lncRNAs involved in the differentiation and maturation of these cells (upon the arrows).

In the following, we will highlight well-established lncRNAs involved in ontogeny and the homeostasis of circulating blood cells and their progenitors (Figure 2).

#### 2.1.1. Erythrocytes 

During red blood cells development, various lncRNAs regulate erythroid gene expression. Although their specific functions are still not elucidated, some critical processes for the homeostasis of erythropoiesis involve pro- and anti-apoptotic pathways. *Fas-antisense 1* (*Fas-AS1*, also known as *Saf*), an lncRNA antisense to *Fas* cell surface death receptor coding gene, was upregulated during the differentiation stage of erythropoiesis by the erythroid transcription factors GATA binding protein 1 (GATA1) and Kruppel-like factor 1 (KLF1), and negatively regulated by the nuclear factor kappa B (NF-κB). In erythroblasts derived from CD34^+^ hematopoietic stem cells, the overexpression of *Fas-AS1* reduced the Fas surface levels and protected cells from death signals [25].

The depletion of 12 out of 13 lncRNAs selected as candidate modulators of erythropoiesis impaired erythrocyte maturation, suggesting that they play crucial roles during late stages of maturation [26]. One of these candidate RNAs is *lincRNA-EPS*, which promotes erythroid differentiation by preventing apoptosis [27].

The lincRNAs *lincRNA-EC2*, *-E4*, and *-EC9* were found to be required for erythrocyte maturation in the foetal liver, but they were absent in the adult bone marrow. The enhancer-derived *alncRNA-EC7* is required for the activation of the neighboring gene *SLC4A1*, which encodes BAND3, a crucial anion transporter of the erythrocyte membrane. The lncRNA *alncRNA-EC7* has an evolutionary conserved enhancer site, and there is evidence of chromatin looping between this site and the *SLC4A1* promoter proximal region, supporting a model whereby *alncRNA-EC7* modulates erythropoiesis. In erythroblasts, the expression of *elncRNA-EC3* is positively correlated with the expression of *KIF2A*, which encodes a microtubule-associated kinesin motor protein, required for mitotic progression. Moreover, the lncRNA *deleted in lymphocytic leukemia 2* (*DLEU2*) is expressed from distinct promoters of *shlncRNA-EC6* in erythroblasts, compared to other cell types, and represses the neighbor gene *SPRYD7*. A deletion in *shlncRNA-EC6* is frequent in B cell malignancies, and *DLEU2* might have an important role in erythropoiesis [26].

The expression of several lncRNAs is restricted to specific stages of erythrocyte differentiation. Often, these lncRNAs are co-expressed with mRNAs and miRNAs in the same stages of cell differentiation. Among the lncRNAs with potential roles during hematopoiesis, *JHDM1D-AS1*, *AC087294.2*, and *RP11-66D17.5* were frequently associated with genes of the erythrocyte mature network, while *RP11-326C3.2*, localized between *NLRP6* and *ATHL1* coding-genes, as well as the co-expressed *ATHL1* both increased during erythrocyte maturation. Moreover, as observed in in silico analysis, the lncRNA *TOPORS-AS1* could act as a ceRNA for three microRNAs involved in the inhibition of erythrocyte development (miR-532-3p, miR-191-5p, and hsa-miR-652-3p) [28]. In a similar previous study, co-expression was found between an lncRNA (not identified by the authors) and miR-4732, a key modulator miRNA during human erythropoiesis and abundant in erythrocytes within the erythroid-enriched miR-144/451 locus, a cluster upregulated by GATA1 during erythrocyte development. This ~250-bp lncRNA overlaps with miR-4732 and is proximal to the miR-144/451 cluster. This cluster has been highly related to erythropoiesis, and all RNAs in this locus were highly expressed in mature erythrocytes, in comparison with RNAs from other genomic regions [29].

#### 2.1.2. Other Myeloid Lineage Cells

Beyond the erythrocytes, the myeloid lineage includes neutrophils, eosinophils, monocytes and dendritic cells, among other important cells for the innate immunity.

The lifespan of short-lived myeloid cells (neutrophils, eosinophils, and Ly6Chi classical monocytes) was found in mice as regulated by the *myeloid RNA regulator of Bim-induced death* (*Morrbid*, also known as *MIR4435-2HG*), in response to pro-survival cytokines. The nuclear lncRNA *Morrbid*, specifically expressed in mature short-lived myeloid cells, neighbors and *cis*-regulates the pro-apoptotic gene *Bcl2l11* (*Bim*) by promoting an H3K27me3 modification at *Bim* promoter through interaction with the polycomb repressive complex 2 (PRC2, a histone modifying complex), repressing the apoptosis in these cells. *Morrbid* deficiency resulted in a drastic reduction of these myeloid cells in blood and spleen, increasing susceptibility to bacterial infection and protecting against eosinophil-driven allergic lung inflammation. Moreover, *MORRBID* dysregulation in humans is associated with hypereosinophilic syndrome, a disorder characterized by severe eosinophilia. Collectively, the lncRNA *Morrbid*, in response to extracellular pro-survival signals, is crucial for the physiologic control of the lifespan of neutrophils, eosinophils, and classical monocytes [30].

Various lncRNAs were highly expressed in monocytes, including the well-known lncRNAs *growth arrest-specific 5* (*GAS5*), *CCAAT/enhancer binding protein β antisense* (*CEBPB-AS1*), *lincRNA P53-induced transcript* (*LINCPINT*), *integrin β 2 complement component 3 receptor 3/4 subunit antisense* (*ITGB2-AS1*), and *FYVE RhoGEF/PH domain containing 5 antisense* (*FGD5-AS1*) [31]. Several other annotated but still uncharacterized lncRNAs were also highly expressed in monocytes, such as *LINC00678*, *LINC00657*, *LINC00989*, and *LINC01420*, which might have a potential role in monocyte regulation. Moreover, novel lncRNAs, such as *LINC01420*, whose gene is located between the *UBQLN2* and *SPIN3* coding genes, and with a moderated expression in other hematopoietic cell types, were also detected in monocytes, apart from the lncRNAs *TCONS_00282615* and *TCONS_00008494* (also known as *lnc-IGFBP7-5*), specific to these cells [31].

During the differentiation of monocytes to dendritic cells (DCs), important epigenetic modifications were found in the promoter region of *HOXA transcript antisense RNA myeloid-specific 1* (*HOTAIRM1*), an lncRNA gene located between *HOXA1* and *HOXA2* genes. *HOTAIRM1* was downregulated in differentiated DCs, and its silencing caused expression changes of several monocyte differentiation markers. Moreover, the upregulation of miR-3960 could downregulate both *HOTAIRM1* and the DC differentiation repression gene *HOXA1*, inducing the differentiation into DCs. The results indicate competitive binding between *HOTAIRM1* and miR-3960, regulating DC differentiation [32].

The *lincRNA-COX2*, neighbor of the *prostaglandin-endoperoxide synthase 2* (*Ptgs2/Cox2*) gene in mice, is highly induced in innate immune response [18,33] and was first described in DCs after toll-like receptor 4 (TLR4) stimulation with lipopolysaccharide (LPS) [18]. Some years later, it was demonstrated that in macrophages stimulated with Pam3CSK4 (a TLR2 ligand), numerous immunological genes were induced, including 62 lncRNAs, of which *lincRNA-COX2* was the most highly induced [33]. In the same study the authors observed that silencing *lincRNA-COX2* did not alter *COX2* expression, but induced 787 genes in unstimulated cells, including chemokines (such as *Ccl5*, *Cx3cl1*), chemokine receptors (such as *Ccrl*), and interferon-stimulated genes (such as *Irf7*). In stimulated cells, *lincRNA-COX2* silencing attenuated the expression of 713 genes, including *Tlr1*, *interleukin (IL-)6*, and *IL-23a*. It was also demonstrated in vitro that *lincRNA-Cox2* interacts with the heterogeneous ribonucleoprotein (hnRNP) A/B and A2/B1, and knockdown of both hnRNPs repressed *Ccl5* and *Irf7* [33]. In contrast, *lincRNA-Cox2* was seen to induce *Ccl5* expression in LPS-stimulated immortalized macrophage cell lines, by recruitment of the chromatin remodeling complex SWI/SNF to the *Ccl5* promoter. Furthermore, *lincRNA-Cox2* is required in the innate response for the expression of NF-κB-regulated late-primary inflammatory response genes [34].

An lncRNA exclusive to DCs is *lnc-DC*, highly expressed from the human *Wdnm1-like* pseudogene at the moment of DC differentiation, driven by PU.1 transcription factor [35,36], being present in Lin^−^MHCII^+^CD11c^+^ conventional DCs, and absent or lowly expressed in CD14^+^ monocytes, B cells, and other hematopoietic cells. The *lnc-DC* binds to the signal transducer and activator of transcription 3 (STAT3) that regulates DC differentiation, protecting STAT3 from inactivation by the protein-tyrosine phosphatase non-receptor type 6 (PTPN6, or SHP1). Therefore, through promoting STAT3 signaling, *lnc-DC* leads to DC differentiation from monocytes to DCs, in vitro and in vivo, and to the DC capacity to activate T lymphocytes [35].

#### 2.1.3. Lymphocytes

Lymphocytes are a class of leucocytes of prime importance for both the innate immunity (innate lymphoid cells—ILCs, in which natural killer—NK—cells are included) and acquired immunity (T and B cells).

Many novel lncRNAs were identified by global gene-expression profiling of human bone marrow and thymic progenitor cells, spanning the earliest stages of B and T lymphoid lineages commitment. These lncRNAs were predominantly intergenic, followed by antisense lncRNAs. The expression of a high proportion of the lncRNAs was positively correlated with that of neighboring protein-coding genes, including genes involved in hematopoiesis and the regulation of lymphocyte development and proliferation. In addition, the authors identified lncRNAs co-expressed with known protein-coding genes specific for different lymphocyte lineages, such as *PAX5*, *EBF1*, *CD3*, *BCL11B*, *NOTCH1*, *RAG1*, and *RAG2*, among many others. The results provide a resource for candidate lncRNA to be further investigated in functional studies in order to clarify their role in lymphopoiesis [37].

Moreover, *NONHSAT040475* (also known as *lnc-BRF1-24*) is an lncRNA widely expressed in human peripheral blood leukocytes. Its expression was compared between T cells (CD3^+^), B cells (CD19^+^), and NK cells (CD56^+^). B cells were the lymphocyte subset with the highest expression of *NONHSAT040475*, in comparison with NK cells and granulocytes, which presented the lowest expression among the analyzed cell types [38].

The next sections will address the lncRNAs associated with specific lymphocyte types.

##### T Lymphocytes

T lymphocytes (or T cells), as one of the central cell types of the immune system, perform crucial roles in adaptive immune responses, which are under constant regulation to maintain body homeostasis. T lymphocytes are subdivided in partially overlapping subgroups based on their more prominent known functions. So, CD4^+^ T helper lymphocytes (Th1, Th2, Th17) are essential for the activation and regulation of B lymphocytes and cytotoxic T lymphocytes (CTL or Tc); CD8^+^ T cytotoxic lymphocytes eliminate virus-infected cells, tumor cells, or otherwise damaged cells; regulatory T lymphocytes (Treg) are essential for immunological peripheral tolerance; and memory T lymphocytes, in their turn, provide a quick response upon re-exposure to their specific antigen. In the first genome-wide characterization of lncRNAs expressed in mammalian (human and mouse) CD8^+^ T lymphocytes, over a thousand lncRNAs were identified, many of which presented stage- or tissue-specific expression, and were mapped close to or overlapped shorter functional RNA or protein-coding genes with quite characterized roles in CD8^+^ T cells [39]. Most lncRNAs expressed in CD8^+^ T cells presented signatures of evolutionary conservation, and their secondary structures and the characteristics of the promoters provided further evidence of functional relevance [39].

Long non-coding RNAs also affect transcription factors present in T cells, as the NFAT complex (nuclear factor of activated T cells). In resting cells, NFAT proteins are located in the cytoplasm, where they are heavily phosphorylated. An increase of intracellular Ca^2+^ concentration activates calcineurin which dephosphorylates the NFAT regulatory domain, ultimately resulting in the nuclear accumulation of NFAT and transcription of NFAT target genes. Phosphorylated NFAT1 is present with the lincRNA *ncRNA repressor of the nuclear factor of activated T cells* (*NRON*) and the multidomain scaffold protein IQGAP1 in a cytoplasmic RNA-protein complex that also contains three known NFAT inhibitory kinases. This supports the hypothesis that lincRNAs may function as scaffolds for transcriptional regulators in large RNA-protein complexes [40]. Long non-coding RNAs can influence Th1/Th2 differentiation by affecting the expression of critical cytokines. The murine lincRNA *Tmevpg1* is required for the control of Theiler’s virus infection, and its human orthologue *TMEVPG1* (also known as *IFNG-AS1*) contributes to interferon gamma expression. The expression of *TMEVPG1* and *IFNG* correlate positively. This lincRNA is preferentially expressed in Th1 cells, depending on transcription factors STAT4 and TBET/T-bet, which drive the Th1 differentiation program [41].

High-throughput RNA sequencing was used to investigate lncRNA expression along the development and differentiation of T cell lineages. Forty-two subsets of thymocytes and peripheral T cells at multiple time points of differentiation were analyzed, and 1524 genomic regions expressing lincRNAs were identified. The effect of *TMEVPG1* in Th1 lymphocyte differentiation (see above) was corroborated, but none of the numerous other lincRNAs had been previously described in T cells. Results revealed highly dynamic stage- and cell-specific expression patterns for lincRNAs during T cell differentiation. Key transcription factors, specific for the Th1 (T-bet and STAT4) and Th2 (GATA3 and STAT6) lineages, were largely responsible for the lineage-specific expression of T cell lincRNAs [42]. A more specific result was obtained for the lincRNA *LincR-Ccr2-5′AS*, which together with transcription factor GATA3 was a fundamental component of the chemokine signaling pathway in Th2 subset cells and important for the migration of these cells [42]. More recently, another 13 subsets of T and B cells were analyzed by RNA sequencing and over 500 previously unknown lincRNAs were identified, suggesting that lincRNAs might collaborate for the determination of lymphocyte identity and for the modulation of their functional plasticity [43]. Beyond that, the influence of *LincMAf-4* (also known as *MAFTRR*) in T cell differentiation was partially clarified. *LincMAf-4* is a chromatin-associated lincRNA specific to the Th1 subset of helper T cells, whose expression was inversely correlated with expression of MAF, a Th2-associated transcription factor. *Linc-MAF-4* regulated *MAF* transcription through the recruitment of chromatin modifiers LSD1 and EZH2; the downregulation of *linc-MAF-4* alters T cell differentiation toward the Th2 phenotype [44]. Furthermore, progenitor cells spanning the earliest stages of B lymphoid and T lymphoid specification were isolated from the bone marrow and thymus for RNA sequencing analysis. More than 3000 genes encoding yet unexplored lncRNAs were described. Highly stage-specific lncRNA expression patterns characterized lymphoid commitment, and several lncRNAs mark the beginning of the divergence of B and T lineages [37]. In addition, more than 2000 lncRNAs were identified in human T cell cultures by RNA sequencing. Moreover, a cluster of antisense lncRNA transcribed from the *RAD50* locus that regulate the expression of Th2 cytokines, IL-4, IL-5, and IL-13 was described [45]. Another Th2 specific lincRNA, *GATA3-AS1*, was analyzed in 27 human tissues and immune cell types, where it was largely undetectable or had very limited expression, supporting the Th2-specific expression pattern and indicating that it might serve as a marker of Th2 response [46]. Microarray analysis of CD4^+^ T cells at different stages also confirmed distinguishable lncRNA expression profiles and highlighted the importance of lncRNAs in CD4^+^ T cell development and activation [47]. Specific lncRNA genes involved in the early differentiation of Th1 and Th2 subsets were identified, and a positive trend in the expression of the lineage-specific lncRNAs and the neighboring protein-coding genes was observed [48].

Long non-coding RNA profiles of naive T cells and two subsets of memory CD4^+^ T cells (central and effector) were obtained by RNA sequencing, and a total of 57,000 novel lncRNAs were found in at least one cell subtype. Many lineage-specific lncRNAs were reported, most of them mapped in a non-random manner, positioned close to the lineage-specific protein-coding genes with which they are co-expressed. Furthermore, it has been demonstrated that lncRNAs mapped near to the *IFNG* locus are necessary for *IFNG* prompt expression, since the expression inhibition of lncRNAs *IFNG-AS1* and *IFNG-R-49* by siRNAs abolishes *IFNG* expression by effector memory T lymphocytes [49].

In view of the huge amount of data generated, especially by high-throughput sequencing technologies, and the recent progress in lncRNA identification, the role of lncRNAs in T cells development as well as in immune responses became evident. Several lncRNAs present a highly lineage-specific expression and help to control the differentiation and function of these important immune cells. Although there is much to explore about T cells and lncRNAs, the importance of these non-coding RNAs is already settled.

##### B Lymphocytes 

B lymphocytes are responsible for the production of immunoglobulins, also known as antibodies, which recognize foreign antigens, and are thus essential for the so-called humoral immunity. Activated B cells can evolve to plasmocytes and memory cells. In a transcriptome analysis of 11 B cells subsets, several lncRNAs expressed along human B cell development and correlated with other RNAs were identified. Among the lncRNAs expressed during the early stage of B cell development, the transcripts *MYB-AS1*, *SMAD-AS1*, and *LEF1-AS1* are antisense to genes of known early B cell transcription factors (*MYB*, *SMAD1*, and *LEF1*, respectively). Another transcript identified was the lincRNA *CTC-436K13.6*, located between the genes *EBF1* (which encodes the transcription factor early B cell factor 1) and *CLINT1*. Several lncRNAs were also identified in proliferative stages and in the germinal center. In the germinal center, the lincRNAs *LINC00487*, *LINC00877*, and *RP11-203B7.2* were found to be highly connected genes (hub genes). Furthermore, the gene for lincRNA, *RP11-132N15.3*, found to be predominantly expressed in centroblasts, is localized upstream of the *BCL6* gene, the main germinal center regulator [50].

In another transcriptome analysis of B cell subtypes of mice, 4516 lncRNAs, were identified as differentially expressed during B cell development and activation, of which 702 were predicted to be associated with enhancers (eRNAs) and 192 with promoter regions (pRNAs). Among them, a strong positive correlation between the eRNA *LNCGme02323* and the cluster lightpink4, which contains 31 protein-coding genes regulated in marginal zone B cells, was observed. Moreover, 784 lncRNAs of pro-B cells and 717 lncRNAs of mature B cells overlapped or were less than 1 kb downstream of binding sites for PAX5, a transcription factor required for B cell development. Evidence of regulation by PAX5 was found for 109 of these lncRNAs, indicating a role for these lncRNAs in B cell homeostasis [51].

Finally, it was recently demonstrated that during female B cell development, the inactivated X chromosome (Xi) loses the heterochromatin modifications and the co-localization within the lncRNA *Xist*, which are required to compensate between sexes for the dosage of X-linked gene products. The lack of *Xist* at the Xi occurs at the pro-B cell stage and persists in non-activated B cells, being restored to Xi during the activation of mature B cells. For restoring of *Xist* localization and chromatin modifications, the transcription factor YY1, involved in V(D)J recombination (variable, diversity and joining gene segments recombination) of B cells, is required. While it remains unknown why the Xi loses heterochromatic modifications and *Xist* RNA during female B cell development, the authors suggested that these features might enable rapid immune-related X-linked genes reactivation in response to infections and could also explain some of the immunological differences between the sexes [52].

##### Natural Killer Cells

Long non-coding RNAs were also seen to be involved in NK cell function and differentiation. In an lncRNA profiling analysis of different populations of NK cells, numerous novel lncRNAs were identified, including *lnc-CD56*, an lncRNA specific to NK cells. *lnc-CD56* positively correlated with the expression of the classical NK cells marker CD56 in primary and some differentiated human NK cells. Moreover, *lnc-CD56* is predicted to interact with the NK-transcription factors TBX21, IRF2, IKZF2, ELF4, and EOMES [53].

A novel killer cell immunoglobulin-like receptor (KIR) gene-body associated antisense transcript not expressed in mature NK cells was described in cells with stem cell properties. The promoter of this spliced lncRNA is at intron 2 of several *KIR* genes, and its sequence is complementary to the proximal promoter of the *KIR* genes and KIR-coding exons 1 and 2. Its promoter contains binding sites for MZF-1, a bi-functional transcription factor that activates transcription in cells of hematopoietic origin and represses transcription in most other cells. In support of a role of that lncRNA in the initial silencing of *KIR* genes, the differentiation of human embryonic stem cells into CD34-expressing hematopoietic progenitor cells was accompanied by the loss of this lncRNA [54].

Moreover, it was recently demonstrated that the *RNA-demarcated regulatory region of id2* (*Rroid*) of mice is an lncRNA locus located ~220 kb from the *id2* gene, a transcriptional regulator that inhibits the expression of T and B cell-specific genes and is essential for lineage commitment and maintenance of mature group 1 innate lymphoid cells (ILC1s). The lncRNA *Rroid* is highly and specifically expressed in ILC1s, where the NK cells are included. In ILC1s, the *Rroid* locus (not the lncRNA itself) directly interacts with the *id2* promoter and, in response to interleukin 15 (IL-15), promotes chromatin accessibility and the deposition of the transcription factor STAT5 at the *id2* promoter, thus acting as a *cis*-regulatory element of the *id2* gene and promoting its expression in ILC1s [55].

### 2.2. Solid Tissues

**Figure 3:**
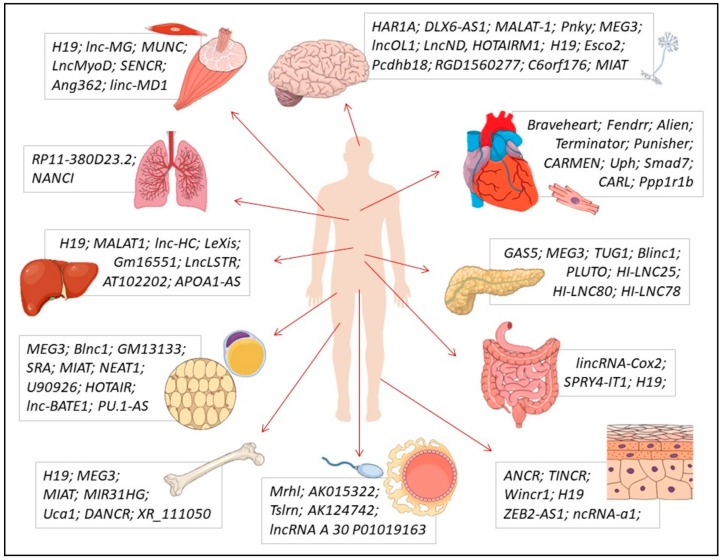
Main lncRNAs described as playing physiological roles (in function, homeostasis, and/or differentiation) in the following (clockwise direction): nervous, cardiac, pancreatic, intestinal, epidermal, germ, bone, adipose, hepatic, lung, and muscular (skeletal and smooth) tissues.

Several lncRNAs were identified in healthy solid tissues and organs, exercising critical roles during their differentiation, metabolism, and physiological functions (Figure 3). These lncRNAs and the regulatory mechanisms that they play in specific solid tissues are reviewed in this section.

#### 2.2.1. Epidermal Tissue 

Epidermal epithelial tissue is a stratified surface epithelium providing a barrier to the external environment. Epithelial cells are closely interconnected (juxtaposed). The epidermis is the outermost layer of the skin and has a high turnover rate due to the continuous shedding of the outer layer of cornified cells. The importance of lncRNAs in epithelial cells has been demonstrated by diverse studies, especially with regard to gene expression regulation [56,57]. One of the first examples is the natural antisense transcript (NAT) *Zeb2-AS1*, an lncRNA that is an important component of the epithelial-mesenchymal transition (EMT) [56]. Among the regulatory proteins required for EMT is the Zeb2 (zinc finger E-box binding homeobox 2) transcriptional repressor of E-cadherin. *Zeb2-AS1* acts to induce the expression of the Zeb2/Sip1 (*ZFHX1B*) gene by promoting the maintenance of a large intron in the 5’ untranslated region (UTR) of the Zeb2 transcript. This region contains an internal ribosome entry site (IRES) necessary for Zeb2 expression, which in turn participates in the downregulation of E-cadherin mRNA and protein levels [56]. Furthermore, several lncRNAs have been identified as differentially expressed during keratinocyte (KCs) differentiation by non-coding RNA array analysis of epidermal equivalents grown in vitro [58], including *AF005081*, *UC003af*, *BC020554*, and *AK022798*, upregulated at different times and differentiation stages. In a study of primary human keratinocytes, lncRNA expression was measured by microarray before and after the induction of differentiation. A total of 687 lncRNAs were detected, of which 104 were differentially expressed following differentiation [59]. The *activating long ncRNA 1* (*ncRNA-a1*, also known as *FALEC*, *focally amplified long non-coding RNA in epithelial cancer*) was found to upregulate *ECM1*, a neighbor protein coding gene, and both the protein and the lncRNA, were induced after keratinocyte differentiation [59]. Other examples are two lncRNAs that control the balance between the progenitor and differentiated compartment of human epidermis: *anti-differentiation non-coding RNA* (*ANCR*, also known as *DANCR*), which is strongly suppressed in differentiated cells, and *terminal differentiation-induced ncRNA* (*TINCR*), which is highly induced during epidermal differentiation [57,60,61]. *Anti-differentiation non-coding RNA* is required to enforce the undifferentiated cell state within epidermis [60], whereas *TINCR* promotes human epidermal differentiation by a post-transcriptional mechanism, mediated by interactions with *staufen1* (STAU1) protein [61]. Moreover, *ANCR* and *TINCR* act as regulators of transcription factors MAF and MAFB, which are important for epidermal differentiation regulation [57]. Interestingly, an lncRNA-miRNA interaction has been reported as influencing keratinocyte differentiation. The lncRNA *H19* can function as a competitive endogenous RNA for miR-130b-3p. This miRNA targets the desmoglein 1 (DSG1) transcript, resulting in decreased DSG1 expression and action, thus keratinocyte differentiation is inhibited. However, lncRNA *H19* adds a regulation point in this differentiation process by acting as an miR-130b-3p sponge and preventing its activity; hence, *H19* can promote keratinocyte differentiation [62]. In addition, lncRNAs, including the aforementioned *H19*, can play a role also in the regulation of dermal papilla (DP) cell function, influencing hair follicle development and regeneration. A comparison of lncRNAs differentially expressed in early- and late-passage DP cells revealed numerous differentially expressed lncRNAs. Three of these, *RP11-766N7.3*, *H19*, and *HOTAIR*, may participate in hair gene regulation by targeting the Wnt signaling pathway, which plays critical roles in skin development and homeostasis and is required for hair follicle initiation [63]. Recently, 111 lncRNAs were shown to be differentially expressed in response to the activation of Wnt/β-catenin signaling in mouse dermal fibroblasts. The Wnt signaling-induced lncRNA *Wincr1* is highly expressed in primary dermal fibroblasts and may affect complex cellular behaviors including collective cell migration and collagen processing and contraction. Also, *Wincr1* affects the expression of several genes, such as *Col1a1* and *Mmp10* [64].

#### 2.2.2. Nervous Tissue

Many lncRNAs are specifically expressed in the brain. These transcripts have been considered essential pieces in the regulation of a broad spectrum of neuronal processes, including brain development, establishment and maintenance of neural cell types, synapse formation and function, stress responses, and age-associated changes [65]. Some lncRNAs have been implicated in brain development, such as *highly accelerated region 1A* (*HAR1A*), which is expressed specifically in Cajal–Retzius neurons in the developing human neocortex from 7 to 19 gestational weeks, a crucial period for cortical neuron specification and migration. *HAR1A* is co-expressed with *reelin*, which is of fundamental importance in specifying the six-layer structure of the human brain cortex [66]. Another example is the lncRNA *DLX6 antisense RNA 1* (*DLX6-AS1*, or *Evf2*), which influences the establishment of murine regional forebrain organization. This lncRNA acts in ventral forebrain development, recruiting DLX and MECP2 transcription factors to DNA regulatory elements in the *Dlx5/6* intergenic region and controlling *Dlx5*, *Dlx6*, and *Gad1* expression through trans and *cis*-acting mechanisms [67]. Moreover, *Pinky* (*Pnky*) is an lncRNA that influences murine neural stem cell (NSC) differentiation. *Pnky* interacts with the PTBP1 protein and regulates neurogenesis from NSCs in the embryonic and postnatal brain [68]. In the Catarrhini branch of primates, the *lncRNA for neuronal development* (*LncND*, also known as *lnc-TMEM18*) is highly expressed in neural progenitor cells and downregulated in neurons. It controls the expression of the Notch signaling pathway, which may be necessary to maintain the stemness of outer sub-ventricular zone radial glia-like cells (oRG), by functioning as a miRNA sponge for the microRNA mir-143-3p. The evolutionary emergence of *LncND* its co-evolution with miRNA-143-3p may have contributed to the expansion of the cerebral cortex in primates [69].

Global RNA sequencing techniques have also been used to investigate lncRNA expression in brain development and function. Neurons derived from induced pluripotent stem cells (iPSCs) have been analyzed by RNA sequencing, and emphatic changes in lncRNA expression during transition from iPSCs to early differentiating neurons were detected. The *HOTAIRM1* (*HOXA transcript antisense RNA myeloid-specific1*) lncRNA is expressed early in neurogenesis, being important for *HOXA* gene regulation during myelopoiesis [70]. Besides, through a microarray approach, 934 lncRNAs were found to be differently expressed when comparing human embryonic stem cells (hESCs) with neural progenitor cells (NPCs). Of these lncRNAs, four were subjected to knockdown that indicated that these lncRNAs are required for adequate neuronal differentiation [71]. Microarray assay was also used to explore the differentiation of bone marrow-derived mesenchymal stem cells (BMSCs) into neural cells. After differentiation induction, 23 lncRNAs were upregulated or downregulated. Further, reverse transcription—quantitative polymerase chain reaction (RT-qPCR) was performed to validate *H19*, *Esco2*, *Pcdhb18*, and *RGD1560277* differential expression, corroborating lncRNAs as key regulators of BMSC differentiation [72]. A transcriptome and splicing database was generated for eight different brain cell types from mouse cerebral cortex: neurons, astrocytes, oligodendrocyte precursor cells, newly formed oligodendrocytes, myelinating oligodendrocytes, microglia, endothelial cells, and pericytes [73]. The expression of 811 lncRNAs was detected and some of them were among the highest expressed transcripts in the mouse brain, with 12 placing among the 7% most highly expressed genes in the RNA dataset. Moreover, some lncRNAs were expressed in cell type-specific or enriched manners [73]. RNA sequencing was also used to profile lncRNA expression in mouse auditory forebrain during postnatal development at time points before and after the onset of hearing [74]. This study revealed that the lncRNA expression profile was distinct between primary auditory cortex and medial geniculate body and between different postnatal time point, indicating spatial and temporal specificity during the maturation of the auditory forebrain [74]. Recently, the human neocortex transcriptome was also analyzed at the single-cell level [75]: hundreds of cells from human neocortex at different stages of development were individually sequenced, revealing that many lncRNAs are abundantly expressed in individual cells and are cell type-specific. For example, *LOC646329*, an lncRNA that regulates cell proliferation, is enriched in single radial glia cells but is detected at low abundance in bulk tissues [75]. In addition, 155 differentially expressed lncRNAs have been reported in a neuronal cell line after stimulation by brain-derived neurotrophic factor (BDNF), a critical neurotrophin for neural development. Long non-coding RNAs with fold changes above 2 included the already known *C6orf176* and *MIAT*; however, many differentially expressed lncRNAs are not currently understood [76]. For oligodendrocytes, a dynamic expression profile at different stages of development was established. Among oligodendrocyte-restricted lncRNAs, the evolutionary conserved chromatin-associated *lncOL1* was functionally explored. Its overexpression promotes oligodendrocyte differentiation in the developing brain, whereas its knockdown inhibited the expression of myelin genes, causing defects in central nervous system (CNS) myelination [77].

Long non-coding RNA are also involved in the regulation of synaptic processes. The nuclear lncRNA *metastasis-associated lung adenocarcinoma transcript 1* (*MALAT1*), also known as *NEAT2* (*nuclear-enriched abundant transcript 2*) or *NCRNA00047*, is highly expressed in neurons and regulates synapse formation by modulating the expression of genes involved in synapse formation and/or maintenance [78]. Furthermore, the lncRNA *maternally expressed gene 3* (*Meg3*) in mice is important for maintaining synaptic plasticity by modulating the phosphatase and tensin homolog (PTEN) / (phosphatidylinoside-3-kinase (PI3K) / (protein kinase B (AKT) signaling pathway. In addition, knockdown of *Meg3* in primary cortical neurons prevents the glycine-induced increase of the GluA1 subunit of AMPARs (α-amino-3-hydroxy-5-methyl-4-isoxazolepropionic acid receptors) in the cell membrane. Thus, *Meg3* is important for modulating AMPAR expression, which mediate fast synaptic transmission in the central nervous system [79].

#### 2.2.3. Muscular Tissue: Cardiac, Skeletal, and Smooth

There are three types of muscle: smooth, skeletal, and cardiac. Recently, lncRNAs have been reported as important regulators of myocyte gene expression, and/or have a role in contraction mechanisms, being involved in muscle development, differentiation, and homeostasis. The cardiac muscle composes the heart, the first functional organ formed during embryo development. The multistep process involved in cardiac myocyte differentiation has been detailed elsewhere (reviewed in [80]) and involves highly coordinated pathways of gene expression modulated by the products of protein-coding and non-coding genes (reviewed in [81]).

The murine heart-associated lncRNA *Braveheart* (*Bvht* or *AK143260*) was one of the first lncRNAs associated with cardiac development. *Bvht* is required for the cardiovascular lineage commitment of nascent mesoderm, through the activation of a core regulatory network of cardiovascular transcription factors, and possibly also mediating epigenetic regulation during cardiomyocyte differentiation by interaction with SUZ12, a component of polycomb-repressive complex 2 (PRC2) [82]. In another study using the mouse model, the mesoderm-specific *foetal-lethal non-coding developmental regulatory RNA* (*Fendrr*) was found to be an activity modulator of two histone-modifying complexes. *Fendrr* binds to the PRC2 and trithorax group/mixed lineage leukaemia (TrxG/MLL) complexes, regulating the epigenetic role of both through the attenuation of the expression of transcription factors that are important in lateral mesoderm differentiation. Thereby, *Fendrr* is essential for normal heart and body wall development [83].

The cardiac-enriched lncRNA *Upperhand* (*Uph*, also known as *HAND2-AS1* or *DEIN*) shares a bidirectional promoter with the *Hand2* gene, which encodes a transcription factor involved in heart development, and is precisely controlled by an upstream super-enhancer locus. *Uph* transcription in cis is required for the permissive epigenetic signature, GATA4 binding, and transcription through the enhancer. Therefore, the loss of *Uph* transcription abolishes *Hand2* expression and development of the heart [84].

Transcriptome analysis of human cardiovascular progenitor cells (CPCs) revealed 570 lncRNAs possibly involved in cardiac differentiation. Among them, the *cardiac mesoderm enhancer associated noncoding RNA* (*CARMEN,* also known as *CARMN* or *MIR143HG*), was one of the most highly expressed. The *CARMEN* lncRNA is upstream of the cardiac mesoderm-specifying gene regulatory network and interacts with the PRC2 components SUZ12 and EZH2. Knockdown of the mouse orthologue (*Carmen* or *AK087736*) inhibited cardiac specification and differentiation of CPCs. Furthermore, *CARMEN* also plays a role in maintaining the cardiac identity of differentiated cardiomyocytes [85].

In a transcriptome analysis searching for ncRNAs differentially regulated during hESC differentiation to vascular endothelial cells, 75 novel lncRNAs whose sequence and functionality might be conserved across vertebrate species were identified. Among them, *ALIEN* (also known as *LINC00261*), *TERMINATOR*, and *PUNISHER* (also known as *AGAP2-AS1*) were functionally characterized in human and other vertebrates’ cardiovascular development. The lincRNA *ALIEN* was expressed in vascular progenitors, and was related to cardiovascular development before vascular or cardiac specialization, being strongly correlated with genes involved in mesoderm and cardiovascular commitment, such as *GATA4*, *EOMES*, *MIXL1*, and *T* (*Brachyury*). Two outcomes were observed after knockdown of *ALIEN*: (1) the downregulation of 503 genes involved in blood vessel development and angiogenesis; (2) the upregulation of 738 genes related to cell adhesion and extracellular matrix remodeling [86]. Moreover, the lincRNA *TERMINATOR*, was expressed in cardiovascular progenitors, and was positively correlated with genes related to cell cycle, DNA repair, and chromatin assembly, and negatively correlated with genes involved in proliferation, regulation, and cell death. *TERMINATOR* was also associated with pluripotency regulators POU5F1 (Oct4), SOX2, ZIC5, and REX1, and its knockdown resulted in the downregulation of 185 genes involved in cell–cell interactions and chromatin remodeling related to pluripotent maintenance. Finally, the antisense lncRNA *PUNISHER*, expressed in mature endothelial cells, was positively correlated with definitive vascular development genes, such as GATA2, and negatively correlated with genes involved in cell cycle regulation, DNA damage response, and chromatin modification. In addition, *PUNISHER*’s knockdown resulted in the decrease of histone H3 phosphorylation and the expression of 802 genes involved in cell division, as well as the increased expression of 831 genes involved in cell adhesion and extracellular interactions, altogether compromising endothelial cell function [86].

In another study in ESCs, 4145 multiexonic lncRNAs (2608 novel) with an enhancer activity were identified during differentiation to cardiomyocytes. One of them was the *SMAD7-lncRNA*, proximal to the *Smad7* gene, and highly expressed in cardiac fibroblasts. *SMAD7-lncRNA* knockdown reduced *Smad7* gene expression, demonstrating the relationship between enhancer-derived lncRNAs and the expression of proximal target genes in cardiac fibroblasts [87].

In a transcriptome analysis of left and right ventricles of neonatal mouse heart, 196 of the 23,949 identified lncRNAs presented dynamic regulation in the cardiac maturation process. In addition, 5% of the 2262 lncRNAs located within 50 kb of protein coding genes were correlated with their neighboring genes’ expression (90.4% positively correlated) [88]. Furthermore, the impact of *Ppp1r1b-lncRNA* on the regulation of its neighboring partner gene *Tcap* was demonstrated. *Tcap* encodes the *Titin Cap* protein that anchors Titin filaments at the Z disk in cardiomyocyte sarcomere organization and myogenesis. Interestingly, the expression ratio of *Ppp1r1b-lncRNA/Tcap* differed between normal cardiac maturation and congenital heart defect phenotypes [88].

In heart metabolism, the first lncRNA identified under physiological conditions was the *cardiac apoptosis-related lncRNA* (*CARL* or *AK017121*). As a sponge lncRNA highly expressed in the heart, *CARL* binds miR-539 that negatively regulates PHB2. Thereby *CARL* maintains the expression of PHB2, supressing the effects of anoxia, such as mitochondrial fission and cardiomyocyte apoptosis [89].

In other muscular tissues, *myogenesis-associated lncRNA* (*lnc-mg*) was recently identified in murine skeletal muscle development and during the skeletal differentiation of muscle stem cells (muSCs). *Myogenesis-associated lncRNA* is enriched in skeletal muscle, and its expression is gradually increased during myocyte differentiation. The authors demonstrated that *lnc-mg* overexpression promotes cell differentiation in vitro, and muscle hypertrophy in vivo. Accordingly, knockout of *lnc-mg* in mice promoted muscle atrophy and loss of muscular endurance. *Myogenesis-associated lncRNA* was shown to function as a ceRNA, acting as a sponge for miR-125b, a negative regulator of insulin-like growth factor 2 (Igf2). Thus, *lnc-mg* overexpression downregulates miR-125b, leading to Igf2 protein increase and enhancing skeletal myogenesis [90].

As already mentioned above, in the *H19/Igf2* imprinted genomic locus is also localized *H19* [91], a trans-regulatory lncRNA also involved in skeletal muscle differentiation and regeneration. *H19* is highly expressed in the majority of embryonic and neonatal tissues, especially in skeletal muscles, and is induced by MyoD, the main regulator of muscle differentiation. The *H19* first exon gives rise to miR-675-3p and miR-675-5p, two conserved miRNAs equally upregulated in skeletal muscle differentiation, which downregulate the DNA replication initiation factor *Cdc6* and the anti-differentiation transcription factors *Smad1* and *Smad5*. Thus, the miRNAs miR-675-3p and miR-675-5p, transcribed within *H19*, repress *Cdc6*, *Smad1*, and *Smad5*, mediating skeletal muscle differentiation and regeneration [88]. Furthermore, *H19* may have a role in controlling the timing of muscle differentiation, as this lncRNA also acts as a sponge for miRNAs of the let-7 family, which are involved in muscle differentiation anticipation and in glucose metabolism in muscle [92].

Another myogenic lncRNA is the *MyoD upstream noncoding* (*MUNC* or *DRR*(*eRNA*)), located 5 kb upstream the *MyoD* transcription start site (TSS), and of which the 5′ end overlaps with the *cis*-acting distal regulatory region (DRR) of *MyoD*. *MUNC* lncRNA induces myoblast differentiation by promoting MyoD association with the DRR enhancer and with the *Myogenin* promoter, and also by increasing the expression of *MyoD*, *Myogenin*, and *Myh3*, as well as the expression of non MyoD-inducible genes [93]. In addition, next to the *MyoD* gene is *LncMyoD*, an lncRNA activated by MyoD during myogenesis and myoblast differentiation. *LncMyoD* binds to IGF2-mRNA-binding protein 2 (IMP2), inhibiting N-Ras and c-Myc mRNAs translation, and thus facilitating cell-cycle exit and promoting terminal differentiation [94].

In the regulation of muscle differentiation, the muscle-specific lncRNA *linc-MD1* acts as a ceRNA in human and murine myoblasts, presenting a decoy activity for two different miRNAs, miR-133, and miR-135. These miRNAs regulate the expression of the transcription factors MAML1 and MEF2C, respectively, which activate the expression of muscle-specific genes. In this way, *Linc-MD1* controls the timing of myoblast differentiation, and when it is overexpressed, the anticipation of the muscle differentiation program occurs [95].

Angiotensin II (AngII) is a hormone involved in vasoconstriction, cellular growth, and other processes. The transcriptome analysis of rat vascular smooth muscle cells (VSMCs) revealed five previously annotated lncRNAs and 24 novel lncRNAs regulated by AngII [96]. Among them, *lnc-Ang362*, an AngII–regulated lncRNA, was identified as the host transcript for miR-221 and miR-222, miRNAs involved in cell proliferation. Knockdown of *lnc-Ang362* leads to the decrease of VSMC proliferation [96]. The *smooth muscle and endothelial cell-enriched migration/differentiation-associated lncRNA* (*SENCR*) was seen in loss-of-function studies as related to decrease of myocardin expression and to the increase of pro-migratory genes expression, which indicates that *SENCR* is a repressor of smooth muscle cell migration and possibly stabilizes the smooth muscle cell contractile phenotype [97].

Finally, it has been discovered that some annotated lncRNAs also contain open reading frames (ORFs) that encode functional peptides, as the case of the muscle-specific lncRNA-derived micropeptide called *dwarf open reading frame* (*DWORF*). *DWORF* has 34 aminoacids, is localized in the sarcoplasmatic reticulum, and enhances the activity of the Ca^2+^ adenosine triphosphatase SERCA calcium pump, enhancing muscle contractility [14]. In this process, SERCA inhibitors are displaced, including the *myoregulin* (*MLN*) [15], another micropeptide encoded by a skeletal muscle-specific putative lncRNA, which has the opposite effect as *DWORF*, inhibiting SERCA calcium pump and reducing muscle performance [15].

#### 2.2.4. Adipose and Bone Tissues

Adipose tissue is composed predominantly of adipocytes, cells specialized in storing energy as fat. White fat (subcutaneous and visceral) is the largest energy reservoir in the body, while brown (muscle-derived) and beige (or brite) fat (white-derived) are critical for thermogenesis, energy homeostasis, and metabolism. Besides these primary functions, adipose tissue also acts as thermal insulation and protection against mechanical shocks. The prevalence of obesity has led to a surge of interest in understanding the mechanisms underlying adipocyte development, as well as the process of differentiation of the pre-adipocyte precursor cells into adipocytes. There is strong evidence indicating that lncRNAs participate in adipogenesis regulation in physiological and pathological contexts.

Global expression patterns of lncRNA have been explored in adipocytes, and 175 lncRNAs were identified as specifically regulated during adipogenesis, a substantial fraction of which are under the control of the same key transcription factors that activate and repress coding genes during adipocyte differentiation [98].

During adipogenesis, most of the identified lncRNAs are involved in and/or bind the peroxisome proliferator-activated receptors (PPARs), a nuclear hormone receptor superfamily which regulates metabolic functions and energy homeostasis. The lncRNA *steroid receptor RNA activator* (*SRA*) has been observed as an adipogenesis promoter, acting in preadipocyte differentiation by associating with PPARc, a master transcriptional regulator of adipogenesis [99]. *NEAT1* is another lncRNA upregulated in adipogenesis, regulating PPARγ2 splicing [100]. *NEAT1* expression is enhanced by *miR-140*, a regulator of developmental pathways and stem cell differentiation, leading to adipogenic phenotype [101]. Finally, lnc-*U90926* decreases 3T3-L1 adipocyte differentiation in mice, via inhibiting the transactivation of PPARγ2 or PPARγ [102].

PU.1 (also known as SPI1) is a transcription factor involved in myelo-lymphoid differentiation and expressed in the adipocyte lineage. In mice, *PU.1* antisense lncRNA (*PU.1 AS*) was shown to promote adipogenesis through preventing *PU.1* mRNA translation via binding to *PU.1* mRNA to form a *PU.1* mRNA/*PU.1 AS* lncRNA duplex in preadipocytes [103].

The lncRNA *HOTAIR* was found expressed in gluteal, but not in abdominal adipose tissue. The ectopic *HOTAIR* expression in abdominal preadipocytes increased the proportion of differentiated cells and the expression of important adipogenic genes, such as *PPARγ* and lipoprotein lipase (LPL, the main enzyme involved with lipid storage in adipocytes). These data indicate that *HOTAIR* expression may underline the heterogeneity of human fat distribution and might be involved in key processes of adipocyte differentiation [104].

Besides that, it has been demonstrated that adipocyte differentiation in brown fat is also regulated by lncRNAs, as *nuclear brown fat lncRNA 1* (*Blnc1*) forms a ribonucleoprotein complex with transcription factor EBF2, promoting brown and beige adipocyte differentiation [105]. This process is enhanced by the heterogeneous nuclear ribonucleoprotein U (hnRNPU or hnRNP-U) assembly, which physically interacts both with *Blnc1* and EBF2, inducing the ribonucleoprotein complex *Blnc1*/EBF2 formation and transcriptional activation of thermogenic genes in brown adipocytes [106].

*Brown adipose tissue–enriched lncRNA 1* (*lnc-BATE1*) was identified in a murine transcriptome analysis as an lncRNA required for the development and maintenance of brown adipocytes. *Lnc-BATE1* sustains the activation of brown adipose related genes, represses white adipose related genes, and interacts with hnRNP-U during brown adipogenesis [107]. A similar lncRNA, *lncBATE10* (*A530050N04Rik*), is also necessary for the expression of brown adipose tissue genes in brown adipocyte differentiation and during the “browning” of white adipocytes. As an effect of the cAMP signaling pathway, *lncBATE10* plays its role by promoting the expression of the transcriptional coactivator Pgc1α, an inductor of mitochondrial biogenesis. Moreover, *lncBATE10* decoys the Pgc1α repressor Celf1, inhibiting their interaction and preventing Pgc1α inhibition [108].

*GM13133* is an lncRNA enriched in murine brown adipocytes and induced by cold exposure and β3-adrenergic agonist and cAMP stimulation. When upregulated, *GM13133* inhibits white adipocyte differentiation and may increase the expression of brown adipose-related genes as well as mitochondrial biogenesis, possibly leading to the conversion of white to brown adipocytes [109]. In addition, 704 upregulated and 896 downregulated lncRNAs were identified in brown adipose tissue when compared with skeletal muscle, again indicating an important role for lncRNAs in adipocyte differentiation, and in transdifferentiation between brown adipose tissue and skeletal muscle, both of which have abundant mitochondria and high energy consumption capacity [110].

Adipose tissues have been also studied in osteogenic processes. During human adipose stem cell (hASCs) differentiation, the expression of the tumor suppressor lncRNA *MEG3* was increased during osteogenesis and decreased during adipogenesis. Accordingly, *MEG3* knockdown blocked the osteogenic differentiation, promoting the adipogenic differentiation of these hASCs. Moreover, the adipogenic and osteogenic balance promoted by *MEG3* during hASC differentiation might be modulated by miR-140-5p, a miRNA negatively correlated with *MEG3* in both processes [111].

Osteogenic differentiation is supressed by cytokine-induced inflammation. Downregulation of the lncRNA *myocardial infarction-associated transcript* (*MIAT*, also known as *Gomafu*) leads to the osteogenic differentiation of hASCs and reverses the negative effects of inflammation. Treatment with tumor necrosis factor increases *MIAT* expression, inhibiting hASC osteoinduction [112]. The lncRNA *MIR31HG* (also known as *LncHIFCAR* or *hsa-lnc-31*) was not only found to be involved in the osteogenic differentiation of hASCs, but also in the regulation of the inflammation. The interaction between *MIR31HG* and NF-κB pathways may attenuate the osteogenic differentiation of hASCs, leading to an inflammation-mediated inhibition of bone formation [113].

As mentioned above, the lncRNA *H19* is a key regulator for the differentiation of various cell and tissue types, and gives rise to miR-675, which is partially responsible for the pro-osteogenic effect of *H19* by downregulating the transforming growth factor-β1 (TGF-β1), inhibiting Smad3 phosphorylation in addition to downregulating histone deacetylase HDAC 4/5, reducing the recruitment of histone deacetylases (HDACs) to the Runt-related transcription factor 2 (Runx2)-binding DNA sequence [114].

Bone morphogenetic protein 9 (BMP9, or GDF2) is one of the most osteoinductive factors. The well-coordinated expression of lncRNA *H19* may be essential in the BMP9-induced osteogenic differentiation of mesenchymal stem cells (MSCs). *H19* is highly expressed at the early stage of the BMP9 stimulation of MSCs, followed by a rapid decease and gradual return to the basal level. Possibly, *H19* functions as a key mediator of BMP9 signaling by modulating miRNAs that target Notch receptors and/or ligands [115].

The RNA profiling of human bone marrow mesenchymal stem cells (hBMMSCs, or hBMSCs) revealed 433 upregulated and 232 downregulated lncRNAs during osteogenic differentiation. The lncRNA *ENST00000502125.2* (also known as *NR2F2-AS1*) was further examined and the results pointed to its role as a negative regulator of osteogenic differentiation, since its downregulation promoted the osteogenic differentiation of hBMMSCs [116]. Global RNA profiling during hBMSC osteogenic differentiation found 785 lncRNAs to be upregulated and 623 lncRNAs downregulated along osteogenic differentiation. Among the differentially expressed lncRNAs were *XR_111050*, *NR_024031*, *FR374455*, *FR401275*, *FR406817*, and *FR148647*. One of them, lncRNA *XR_111050*, could enhance the osteogenic differentiation potential of MSCs [117].

Furthermore, the lncRNA *urothelial cancer-associated 1* (*UCA1*) was found to be expressed in human normal chondrocytes and induced chondrocytic differentiation in a murine chondrocyte precursor [118]. Still during osteogenic differentiation, the lncRNA *differentiation antagonizing non-protein coding RNA* (*DANCR*, also known as the aforementioned *ANCR*) was decreased in human bone marrow-derived mesenchymal stem cells (hBMSCs). When silenced, the proliferation of hBMSCs and osteogenic differentiation is significantly enhanced, and vice versa. This negative control of osteogenic differentiation and proliferation by *DANCR* is achieved through the inactivation of the p38 MAPK pathway, and *DANCR* is suggested by the authors as a potential therapeutic target [119].

#### 2.2.5. Hepatic Tissue

The liver is the largest internal organ of the body, as well as an exocrine and endocrine gland. Like adipose tissue, the liver is responsible, among other important functions, for adipogenesis and lipid metabolism. The lncRNAs involved in liver homeostasis are shown in this section.

During liver development, the lncRNA *H19* was found in a chromatin immunoprecipitation sequencing (ChIP-seq) analysis to be the most highly expressed lncRNA. *H19* blocks the interaction between actin and the heterogeneous hnRNP-U, affecting the Wnt/β-catenin signaling pathway and the proliferation of foetal liver cells [120].

In liver metabolism, the NAT lncRNA *APOA1-AS* negatively regulates *apolipoprotein A1* (*APOA1*) gene expression by epigenetic mechanisms. It was demonstrated, both in vitro and in vivo, that knockdown of *APOA1-AS* alters certain histone methylation patterns through the recruitment of histone-modifying enzymes, increasing the expression of the closely linked *APOA1*, *APOA4*, and *APOC3* genes [121]. The process of cholesterol efflux and the inhibition of cholesterol biosynthesis in the liver is promoted by the ligand activation of liver X receptors (LXRs) and mediated by the lncRNA *LeXis*. *LeXis* can be induced by pharmacologic LXR activation and Western diet feeding, and interacts with ribonucleoprotein RALY, required for cholesterologenic gene expression, thus affecting hepatic and plasmatic cholesterol levels in mice [122].

*Liver-specific triglyceride regulator* (*lncLSTR*) regulates plasmatic triglycerides (TG) in mice. The knockdown of *lncLSTR* leads to the downregulation of Cyp8b1, altering the ratio of the most abundant bile acids, muricholic acid and cholic acid. This alteration results in the upregulation of apoC2 expression and lipoprotein lipase (LPL) activity, increasing farnesoid X receptor (FXR) activity (an important bile acid receptor) and plasmatic TG clearance. However, the increased expression of apoC2 in primary hepatocytes did not lead to the same effects of TG regulation, indicating the presence of other hepatic factors involved in plasmatic TG clearance [123].

A novel sense-overlapping lncRNA named *lnc-HC* was identified in rats, and homologues exist in mice and humans. It overlaps the predicted 3′UTR region of the coding gene *Slc25a15* (mitochondrial carnitine transporter). *Long non-coding RNA derived from hepatocytes* (*lnc-HC*) is a negative regulator of cholesterol metabolism and is upregulated in hepatocytes through C/ERBb when exposed to high cholesterol. This lncRNA physically interacts with heterogeneous nuclear ribonucleoprotein A2/B1 (hnRNPA2B1), forming the *lnc-HC*/hnRNPA2B1 complex, which targets the mRNA of *Cyp7a1* and *Abca1*, decreasing their protein levels and, consequently, downregulating bile acid biosynthesis and cholesterol export [124].

Among the 359 tissue-specific lncRNAs expressed in metabolic homeostasis and found in the transcriptome analysis of murine major metabolic organs, the liver metabolically sensitive lncRNA *Gm16551* was identified. The findings support a model in which SREBP1c, the master transcription factor of lipogenesis, induces *Gm16551* to activate an lncRNA-mediated negative feedback loop to regulate SREBP1c activity in the liver. The *Gm16551* knockdown led to the upregulation of key lipogenic genes and to high circulating triglyceride levels [125].

The benefits of green tea in cholesterol metabolism, due to the high levels of catechins—mainly epigallocatechin-3-gallate (EGCG)—are well-known, but the molecular mechanisms underlying this effect are incompletely understood. Microarray analysis of RNA expressed in cultured human hepatocytes (HepG2) revealed 15 genes related to cholesterol metabolism and 285 lncRNAs whose levels were altered by EGCG treatment. Among them, the lncRNA *AT102202* mapped within the gene of its potential target, 3-hydroxy-3-methylglutaryl coenzyme A reductase (HMGCR). HMGCR is the rate-regulating enzyme in the cholesterol biosynthetic pathway. EGCG treatment enhanced *AT102202* expression and decreased the expression of HMGCR. The downregulation of HMGCR expression leads to LDL receptor upregulation and cholesterol uptake by hepatocytes [126].

In liver regeneration, it was found in vitro that the lncRNA *MALAT1* (or *NEAT2*), upregulated in this process by p53, stimulates cell cycle progression from G1 to S phase and inhibits apoptosis, leading to hepatocyte proliferation. *MALAT1* also activated the Wnt/β-catenin pathway and promoted cyclinD1 expression. These data indicate the critical role of *MALAT1* in liver regeneration, and its potential therapeutic benefit in liver failure and transplantation [127].

#### 2.2.6. Pancreatic Tissue

The pancreas is both an endocrine and an exocrine gland, producing digestive enzymes delivered to the duodenum through the pancreatic duct, and endocrine hormones secreted into the blood, including insulin and glucagon produced, respectively, by β and α cells in the islets of Langerhans. lncRNAs have been studied in pancreatic β cells, due to the importance of these cells for glucose homeostasis and clinical relevance in certain disorders such as diabetes.

More than 1100 lncRNAs were identified in a transcriptome analysis of human pancreatic islets, of which various were specific to β cells and located in genomic regions close or antisense to coding genes involved in the development, expression, and function of β cells, such as *HNF1A*, *PDX1*, *PAX6*, *ISL1*, *FOXA2*, *NEUROD1*, *GATA6*, and *MAFB*. Among them, the expression of the lincRNA *HI-LNC25*, which shares a regulatory domain with the islet cell maturation regulator *MAFB*, was positively correlated with the mRNA levels of GLIS3, an important transcription factor for insulin biosynthesis. Other lncRNAs were upregulated by glucose exposure, such as *HI-LNC78*, *HI-LNC80*, and the murine lncRNA *Mi-Linc80* [128]. *HI-LNC15* (also named *β-cell long intergenic noncoding RNA 1*, *βlinc1*) was further investigated [129]. It is a conserved lncRNA whose gene is located upstream to the islet homeobox transcription factor gene *NKX2.2*, and it is specific to embryonic pancreas and adult islets, especially β cells. *βlinc1* knockdown led to insulin resistance in adult mice and, in embryonic pancreas, to a 50% reduction in β cells of perinatal mice. Moreover, *βlinc1* deficiency dysregulated islet transcription factors, such as Nkx2.2, Pax6, Mafb, and Neurog3, and several genes involved in the specification of endocrine progenitors and in β cell maturation and function [129].

Furthermore, human β cell-specific lncRNAs and transcription factors were found to regulate a common transcriptional network in β cells. These lncRNAs were involved in the regulation of enhancer clusters. Among them, the lncRNA *HI-LNC71*, named by the authors as *Pdx1 locus upstream transcript* (*PLUTO*), is transcribed from a promoter located upstream from the key β cell transcription factor gene *PDX1*. *PLUTO* controls the transcription of *PDX1* and, thereby, modulates the PDX1-dependent transcriptional program. The regulation of *PDX1* by *PLUTO* is associated with its ability to promote three-dimensional (3D) interactions between the *PDX1* promoter and its upstream enhancer cluster, which is contained within the body of the *PLUTO* gene [130].

It was shown in a mouse model that the expression levels of several transcription factors, including Pdx1 and the β cell-specific transcription factor MafA, were reduced after knockdown of lncRNA *Gas5* (*growth arrest-specific transcript 5*), or of the *mouse maternal expressed gene 3* (*Meg3*), or also of the *taurine upregulated gene 1* (*TUG1*). These lncRNAs are highly expressed in pancreatic islets and their expression is supressed at higher glucose concentrations and in diabetic mouse models. In addition, *Gas5* downregulation induced cell cycle G1/G0 arrest, blocking β cell proliferation, while *Meg3* or *TUG1* suppression increased the rate of β cell apoptosis in vitro. Taken together, the lncRNAs *Gas5*, *Meg3*, and *TUG1* might be involved β cell identity and function by regulating transcription factor levels, thereby influencing insulin synthesis and secretion [131,132,133].

In pregnancy, prolactin signaling stimulates β cell proliferation to increase the cell mass in response to greater insulin resistance. During the pregnancy stage corresponding to the peak of β cell proliferation in the mouse (14.5 days), six lncRNAs were found to be differentially expressed in pancreatic islets. Among them, the lncRNA *Gm16308* (*Lnc03*) was prolactin-dependent, islet-enriched, and dynamically expressed at different pregnancy stages. *Lnc03* is regulated by Stat5-dependent components of the prolactin activation pathway. Its expression peaked at gestational day 14.5, but was low before pregnancy and post-partum. Interestingly, the *lnc03* human orthologue was not found. Therefore, *lnc03* may belong to the set of non-conserved lncRNA and the human lncRNA functionally corresponding to *lnc03* still remains unknown [134].

#### 2.2.7. Intestinal Tissue

In intestinal mucosa, the epithelial cells form a single-cell layer that constitutes the largest and most important barrier protecting the intestinal lumen against intraluminal toxins, antigens, and enteric flora. The epithelium maintains its selective barrier function through the formation of complex protein-protein networks that mechanically link adjacent cells and seal the intercellular space.

Among the few studies investigating lncRNAs in intestinal tissue, it was demonstrated that the lncRNA *SPRY4-IT1* increases the stability and translation of the mRNA encoding tight junction (TJ) proteins claudin-1, claudin-3, occludin, and JAM-1. Accordingly, the silencing of *SPRY4-IT1* repressed the expression of these TJ proteins in the intestinal epithelial barrier. The direct interaction between *SPRY4-IT1* and TJ mRNAs is enhanced by the RNA-binding protein HuR. Patients with some intestinal diseases presented lower levels of *SPRY4-IT1*, suggesting that decreased *SPRY4-IT1* and the resulting lower expression of TJ proteins contribute to the pathogenesis of gut barrier dysfunction in patients [135]. Also regulating homeostasis of the intestinal epithelial barrier, the lncRNA *H19* may act as a ceRNA, competing with the miR-874, a microRNA which supresses the expression of the aquaporin-3 gene (*AQP3*) by binding to the 3′UTR of its mRNA. *AQP3* downregulation opens the TJ complex, leading to dysfunction of the intestinal barrier. *H19* competes with miR-874, reducing its levels and maintaining normal levels of AQP3, therefore playing a protective role in the intestinal barrier [136]. However, the overexpression of *H19* and the consequent increase of miR-675 inhibited the synthesis of the TJ protein ZO-1 and the adherens junction protein E-cadherin, leading to intestinal epithelial barrier dysfunction. Epithelial barrier normal function was restored by an increase of the expression of HuR (also known as ELAVL1) in these cells [137].

A genome-wide transcriptome analysis of murine intestinal epithelial cells showed that tumor necrosis factor alpha (TNF-α) stimulation up- and downregulated several lincRNAs genes. Among them, *lincRNA-Cox2* was upregulated in an early response to TNF-α, through the NF-ĸB signaling pathway activation. *lincRNA-Cox2* knockdown altered the gene expression profile in response to TNF-α stimulation. It was found that *lincRNA-Cox2* regulates the transcription of the proinflammatory cytokine interleukin 12b gene (*Il12b*) through epigenetic mechanisms, indicating a role of this lincRNA in the inflammatory responses of the intestinal epithelia [138].

#### 2.2.8. Lung Tissue

The lungs are the primary organs of the respiratory system. Their major function is gas exchange between the air and the blood. They supply oxygen to be distributed to tissues and expel carbon dioxide that has been created throughout the body. Studies reporting the roles of lncRNAs in lung development and homeostasis are still limited.

A total of 363 lncRNAs have been identified in developing and adult lung by an RNA sequencing approach. Spatial correlation between the lncRNAs loci and transcription factor loci across the genome was reported. In addition, their expression pattern correlates with that of the neighboring genes, suggesting the involvement of these lncRNAs in the regulation of the transcription factor genes. In fact, a feedback loop has been identified within the *NANCI-Nkx2.1* gene duplex that is critical for buffering the expression of the transcription factor Nkx2.1, maintaining lung epithelial cell identity during development and postnatal tissue homeostasis: while Nkx2.1 directly inhibits the expression of the lncRNA *NANCI (Nkx2.1-associated noncoding intergenic RNA,* also called *LL18*), *NANCI* acts in cis to promote Nkx2.1 transcription [139,140].

Moreover, a novel lncRNA *RP11-380D23.2* has been reported to drive distal-proximal lung morphogenesis by regulating its nearest gene *PITX2*, which encodes a transcription factor crucial for maintaining the left-right symmetry during lung development. The lncRNA *RP11-380D23.2* modulates *PITX2* via physical interaction with PARP1, a nuclear protein that functions as a chromatin modulator. The *RP11-380D23.2*–PARP1 interaction orchestrates lung development by regulating the timely activation of the Wnt cascade that is important for lung morphogenesis [141].

### 2.3. Gametogenesis and Germ Cells

Gametogenesis is a highly regulated complex cellular differentiation process. The accurate development of oocytes and sperm is among the most fundamental processes for the success of sexual reproduction. Similar to other reproductive traits, many features of gametogenesis and especially of spermatogenesis are under strong selective pressure to change. However, some fundamental features of gametogenesis are evolutionary conserved. Moreover, among all tissues, the transcriptome of the testis displays the highest diversity and specificity.

The lncRNA/messenger RNA gene pair *AK124742* and *PSMD6* presents elevated expression in human cumulus cells, the cluster of cells that surrounds the oocyte, and are correlated with oocyte maturation, fertilization, embryo quality, and clinical pregnancy outcome [142]. Furthermore, a miRNA-lncRNA analysis indicated 41 lncRNAs able to interact with oocyte microRNAs and potentially involved in the regulation of folliculogenesis [143]. In a study that investigated the role of lncRNAs in mediating the effects of β-crystallin B2 (CRYBB2) in ovary development in mice, RNA microarray profiling showed 157 differentially expressed lncRNAs and 1085 differentially expressed mRNAs between ovary tissues from wild type and CRYBB2 knockout mice. In CRYBB2 knockout mice, the expression of lncRNA *A-30-P01019163* was downregulated, as well as its likely downstream target gene *P2rx7*, affecting ovarian development [144].

Concerning mammalian spermatogenesis, the analysis of the Gc1-Spg cell line, derived from mouse spermatogonial cells, demonstrated that the lncRNA *meiotic recombination hot spot locus* (*mrhl*) negatively regulates the Wnt signaling pathway (implicated in the regulation of mammalian spermatogenesis) through Ddx5/p68 RNA helicase [145]. Furthermore, genes at 37 loci (referred to as GRPAM) also showed altered expression upon *mrhl* downregulation. The p68 helicase interacted with *mrhl* in chromatin at these loci, suggesting a role for p68-mediated *mrhl* RNA occupancy in regulating GRPAM expression [146]. Moreover, a functional study reported that the lncRNA *AK015322* promotes the proliferation of the mouse spermatogonial stem cell line C18-4 in vitro by function as a decoy of miR-19b-3p. *AK015322* antagonizes the function of this microRNA, thus indirectly upregulating its endogenous target transcriptional factor Ets-variant 5 (ETV5), which is a pivotal gene for spermatogonial stem cell self-renewal [147].

Long non-coding RNA profiling in mature mouse cells led to the identification of 20,907 known and 4088 novel lncRNAs. Compared to round spermatids, 1794 lncRNAs were upregulated and 165 downregulated, and 14,259 potential target coding-genes of differently expressed lncRNAs were suggested [148].

Purified cells at key stages of murine male spermatogenesis were submitted to RNA sequencing. The dynamic expression pattern of lncRNAs during the progression of spermatogenesis was reported, where 1630 lncRNAs differently expressed in spermatogonia versus pachytene and 1534 lncRNAs differently expressed in pachytene versus spermatids were identified. To examine the possible role of one lncRNA, a mouse knockout of the X-linked lncRNA *testis-specific long noncoding RNA 1* (*Tslrn1*) was generated. These mice had normal fertility but expressive reduction in sperm count [149].

### 2.4. Extracellular Vesicles 

Extracellular vesicles (EVs) play an important role in intercellular communication through the transference of their cargo between cells. They are sub-micron membrane vesicles released to the extracellular environment by all cell types. According to their biogenesis, membrane, size, and composition, EVs can be classified into exosomes, apoptotic bodies, microvesicles, and ectosomes, among others (reviewed in [150,151]). EVs have been found in body fluids, including blood and urine. They are composed of a lipid bilayer from the plasma membrane and contain a variety of proteins, nucleic acids, lipids, and metabolites from donor cells, transferring biomolecular information between tissues and mediating diverse biological functions. Accordingly, RNA transcripts contained in the EVs also may be transferred to the target cells and present functional impact [152,153]. The number of studies concerning lncRNAs in EVs is steadily increasing. Most aim at finding potential disease markers or at understanding pathological mechanisms of human diseases. Yet some of the findings are useful also for the comprehension of physiological processes.

Interestingly, lncRNAs with low expression in cells may be enriched in EVs, such as *lincRNA-p21*, *HOTAIR*, and *ncRNA-CCND1* [154,155]. In fact, in a study comparing normal cell lines with a prostate cancer cell line, the population of exosomal lncRNAs differed between both cell lines. Furthermore, some lncRNAs enriched in EVs present complementary motifs to some microRNAs, such as members of the let-7 family, among others, suggesting that these lncRNAs can act as sponges for EV microRNAs, and to distinct RNA-binding proteins, such as ELAVL1 (HuR), which may play a role in the exosomal transport of lncRNAs [155]. Another functional study provided evidence that specific lncRNAs are preferentially packaged into exosomes, and some of these exosomal lncRNAs (such as *Exo2*, *Exo4*, and *RMRP*) can modulate protein functions and cell viability by direct interactions with l-lactate dehydrogenase B (LDHB), high-mobility group protein 17 (HMG-17), and CSF2RB, inside the recipient [156].

In a total transcriptome profiling of plasma-derived EVs of five healthy women, thousands of different transcripts were found. Among these, up to 220 lncRNA were detected. The lncRNAs *RN7SL1*, *RPPH1*, *VTRNA2-1*, *RMRP*, *RNU11*, and *MALAT1* were the most highly expressed [157]. To our knowledge, this is the first study characterizing EV-lncRNA exclusively in healthy individuals. Thus, the EV-lncRNA cargo remains largely unknown, and the need for more research to characterize EVs contents and biological effects becomes evident. Apart from the relevance of such studies for learning more about extracellular lncRNAs (and other biomolecules) in physiological settings, there is great interest in developing EVs analysis for “liquid biopsies”, which are minimally invasive assays that will be of value in biology and medicine.

## 3. Conclusions

Although lncRNAs were overlooked for a long time, now their importance in various biological processes has become evident. However, their description and especially their effects and functions in homeostasis (and in disease) remain largely elusive. This is not surprising in view of the complex interactions among the multiple players involved in every biological process, and the limitations of the available biological materials and methods. These limitations certainly will be overcome by the improvement of systems biology approaches in wet laboratories as well as bioinformatics, and the development of more powerful integrative analysis methods. In this review, we provided a broad overview of the information available in the literature regarding lncRNA identification and/or effects in specific mammalian—and especially human—cell and tissue types, aiming at understanding their effects in homeostasis. We limited our search to cells and tissues for which there is presently growing interest among researchers. Future studies will expand the knowledge about lncRNAs in cell biology and physiological processes, contributing to a deeper understanding of their function in the context of the multiple interactions between lncRNAs, other ncRNAs, mRNAs, proteins, and small molecules. Furthermore, information about lncRNAs in homeostasis provides a framework for future studies of their functions in health and disease and in the development of novel disease markers and therapeutic targets.

## Supplementary Material

The following are available online at http://www.mdpi.com/2311-553X/4/1/3/s1, Table S1: lncRNAs described in the homeostasis of different cells and tissues.
